# Web‐Based Cognitive Rehabilitation for Patients With Aggressive Lymphoma With Perceived Cognitive Impairment: A Randomised Pilot Study

**DOI:** 10.1002/pon.70469

**Published:** 2026-04-21

**Authors:** Priscilla Gates, Jade Guarnera, Karla Gough, Haryana M. Dhillon, Heather J. Green, Michael Dickinson, Janette L. Vardy, Tracey Dryden, Patricia M. Livingston, Juan Dominguez Duque, Karen Caeyenberghs

**Affiliations:** ^1^ School of Psychology Deakin University Burwood Victoria Australia; ^2^ Centre for Health Services Research in Cancer Peter MacCallum Cancer Centre Parkville Victoria Australia; ^3^ Sir Peter MacCallum Department of Oncology The University of Melbourne Parkville Victoria Australia; ^4^ Department of Nursing The University of Melbourne Parkville Victoria Australia; ^5^ Psycho‐Oncology Cooperative Research Group The University of Sydney Sydney New South Wales Australia; ^6^ Griffith University Gold Coast Queensland Australia; ^7^ Clinical Haematology Peter MacCallum Cancer Centre and Royal Melbourne Hospital Parkville Victoria Australia; ^8^ Concord Cancer Centre Concord Repatriation and General Hospital Concord West New South Wales Australia; ^9^ Faculty of Health Centre for Quality and Patient Safety Research in the Institute for Health Transformation Geelong Victoria Australia

**Keywords:** aggressive lymphoma, cancer‐related cognitive impairment, cognitive rehabilitation intervention, pilot randomised controlled trial

## Abstract

**Objective:**

Cancer‐related cognitive impairment (CRCI) is a frequent side effect of cancer and its treatment that can persist well after treatment completion, with major impacts on quality of life, daily living activities, and return to work. This randomised pilot study assesses the feasibility and acceptability of methods and procedures intended for use in a definitive trial of a web‐based cognitive rehabilitation program—‘Responding to Cognitive Concerns” (eReCog)—in people with low perceived cognitive function after chemotherapy for aggressive lymphoma within the past 5 years and were in remission. Potential efficacy was also explored.

**Methods:**

Participants were randomised one‐to‐one to receive usual care or eReCog plus usual care. The 4‐week eReCog program consists of four online modules based on the principles of cognitive behavioral therapy. Operational, neuropsychological test and patient‐reported outcome measures (PROMs) data were collected before randomisation and approximately 8 weeks later to assess trial outcomes. Primary feasibility outcomes included recruitment and retention rates (a priori progression criteria: ≥ 3 patients/month and ≥ 80% of participants complete the trial, respectively). Feasibility data were summarised using a rate or proportion, as appropriate, with 95% confidence intervals. Neuropsychological test and PROMs data were analyzed using analysis of covariance.

**Results:**

38 of 53 eligible participants consented to participate over 10 months (3.8 patients/month, 95% CI [2.7, 5.2]), 19 were randomised to each arm, and 36 of 38 (95%, 95% CI [83, 99]) completed the trial, indicating acceptable feasibility. Acceptable feasibility was also found for all four secondary outcomes: adherence to, usability of, and intrinsic motivation to engage with eReCog; and compliance with assessments. A large‐sized difference favoring the intervention arm was observed on the SCWT Word score measuring processing speed. Medium‐sized differences were observed on other neuropsychological test and PROM scales, but confidence intervals were wide and included zero.

**Conclusions:**

Recruitment and retention rates, compliance with assessments and favorable changes on potential outcome measures suggest a large‐scale, appropriately powered trial is warranted, as do findings that eReCog is acceptable to the study population.

**Trial registration number:**

Australian New Zealand Clinical Trials Registry ACTRN 12623000705684

## 1 Introduction

Improvements in treatment for cancer have increased long‐term survival rates, resulting in greater numbers of individuals living with long‐term side effects of cancer therapies [1]. Cancer‐related cognitive impairment (CRCI) is a frequent side effect of cancer and its treatment that can persist well after treatment completion [2], with major impacts on quality of life, daily living activities, and return to work [2, 3].

Persistent changes in cognitive performance are frequently reported by survivors of aggressive lymphoma [4, 5, 6, 7, 8, 9, 10, 11]. Our previous study demonstrated that people undergoing standard chemotherapy for aggressive lymphoma performed worse on measures of information processing speed, executive function, and learning and memory both before and after chemotherapy compared to healthy controls [11]. Our longitudinal study in the same population also suggested that cognitive impairments, as measured by objective and subjective cognitive measures, were apparent before chemotherapy [6]. The impact of the high incidence of CRCI; is heightened by a lack of knowledge about how best to support people experiencing this problem [3].

Cognitive rehabilitation refers to a set of therapeutic interventions aimed at improving impaired cognitive functions [12]. The goal is to help individuals restore or compensate for lost cognitive abilities. Cognitive rehabilitation is an umbrella term for interventions directed at one or more cognitive targets such as strategy training (e.g., psychoeducation and skill development to address psychosocial, emotional, and behavioral impacts) and/or cognitive training (e.g., computerised working memory training) [12]. Web‐based interventions offer many advantages to face‐to‐face programmes as they serve a wider demographic group, are cost‐effective, provide participants with the flexibility and convenience to complete tasks individually in their home and offer anonymity [13, 14]. Cognitive rehabilitation can lead to improvements in subjective and multiple objective cognitive functioning domains in adult cancer survivors across a range of central nervous system (CNS) and non‐CNS cancer diagnoses, as demonstrated in our recent systematic review [12]. Moreover, differential effects on specific cognitive domains (e.g., processing speed) and subjective cognition was observed for different interventions [12]. For example, Mihuta et al. (2018) reported those receiving a strategy training web‐based cognitive rehabilitation program (“Responding to Cognitive Concerns,” eReCog) improved perceived cognitive functioning and executive functioning compared to the waitlist group [13].

Further, the review highlighted that most cognitive rehabilitation studies (*n* = 20; 38%) focus exclusively on people with breast cancer; followed by mixed cancers (*n* = 14; 27%), and CNS cancers (*n* = 12; 23%), only seven (13%) included people with lymphoma [12]. In our previous study using a web‐based cognitive training program (i.e., Brain HQ from Posit Science) focused on attention, memory and navigation, we showed an improvement in perceived cognitive abilities, learning, and processing speed post‐intervention in 32 people following autologous bone marrow transplantation including lymphoma (37%) [8, 12]. The web‐based cognitive rehabilitation program “Responding to Cognitive Concerns” (eReCog) addresses multiple domains of cognition, strategy training, and psychoeducation on topics such as fatigue and emotional changes associated with cancer and treatment [14]. Specifically, we used eReCog, a web‐based cognitive rehabilitation program that offers strategy training to educate adult cancer survivors on aging, health, cancer and cognitive function; memory and attention; and fatigue and emotions [14]. In our systematic review, no cognitive rehabilitation studies addressed fatigue, and only two addressed anxious symptomatology, which often co‐exist with CRCI [12].

Here, we aimed to test the feasibility and acceptability of methods and procedures for trialling a web‐based cognitive rehabilitation program (eReCog), in this population. Our secondary aim was to obtain preliminary evidence of efficacy potential of eReCog. Our objectives were to estimate recruitment and retention rates (primary outcomes), and to evaluate adherence to, usability of, and intrinsic motivation to engage with eReCog, compliance with assessments, and potential efficacy.

## 2 Methods

### 2.1 Study Design and Setting

The present study was a single‐site, parallel‐group, pilot randomised controlled trial, with one baseline and one follow‐up assessment (i.e., before randomisation and approximately 8 weeks later). It was conducted in a clinical haematology service at a specialist cancer center in metropolitan Melbourne, Victoria, Australia. Study methods and procedures are described in detail in the published protocol [15], and summarised below.

### 2.2 Study Population

Individuals were considered eligible if they were 18 years or older; had completed chemotherapy for aggressive lymphoma within the past 5 years and were in remission at enrollment; and had a perceived reduction in cognitive functioning based on the single‐item Cognitive Change Screen (i.e., a score of 28.5 or higher); shown to have sensitivity and specificity in people with mixed cancer diagnoses [16]. Eligible individuals had access to a computer with internet and an active email account; were able to read and comprehend English; and had an ECOG Performance Status score of ≤ 2 [17]. Individuals meeting the following criteria were excluded: lymphomatous central nervous system (CNS) involvement; prior cranial radiotherapy; CAR T‐cell therapy or allograft; a life expectancy of less than 12 months; any medical condition that could compromise adherence to study procedures or lead to prolonged hospitalisation; and/or a documented history of past/current substance over use or poorly controlled psychiatric illness.

### 2.3 Recruitment Procedures

Eligible individuals were approached and invited to participate by the clinical team (MD, TD). A copy of the participant information statement and consent form (PICF) was provided and written informed consent was obtained from all participants.

### 2.4 Randomisation

Participants were randomised one‐to‐one to usual care (control) or “Responding to Cognitive Concerns” (eReCog) plus usual care (intervention) following baseline assessment. Randomisation was performed using a computer‐generated block allocation sequence and carried out by a member of the research team not involved in assessment of participants or delivery of intervention.

### 2.5 Interventions

Usual care consisted of a Cancer Council Australia factsheet, “Understanding Changes in Thinking and Memory: A guide for people affected by cancer.” [18] The intervention consisted of the Cancer Council Australia factsheet and access to the web‐based eReCog program. The program is based on the principles of cognitive behavioral therapy and offers psychoeducation on cognitive impairment associated with cancer and its treatment, skills training for improving memory and attention, and relaxation training. It consists of four discrete modules: aging, health, cancer and cognitive function; memory; attention; and fatigue, emotions, and cognition [14]. Participants are expected to complete one module per week over 4 weeks. Modules comprise 13–20 activities (e.g., goal setting, problem solving, attention and memory, strategies and application, and self‐care) and take 30–60 min to complete [14]. The eReCog program included deidentified on‐line interactive pages where reflection exercises, asynchronous group discussion, and weekly online homework tasks were available. It was supported by a program facilitator via email exchange (see below for more information), who followed the clinician manual [19]. The facilitator tracked progress through the modules to ensure participants completed each module before access to the next was enabled.

### 2.6 Engagement With eReCog and Program Satisfaction

Engagement with eReCog was calculated for each of the four modules by assessing the participants' number of responses entered in the free text boxes throughout modules. Participants' responses were reviewed for quality/accuracy by the researchers, with arbitrary text responses (such as non‐words or blank responses) obtaining zero points for that activity. A total engagement score was calculated by summing the points of the activities across the four modules, resulting in a maximum possible score of 76 points [14].

The number of emails received and sent from the program facilitator (JG) were summed as an additional measure of engagement with eReCog. A minimum of eight emails was required to provide links to each of the four modules. Emails were classified based on their content including introducing eReCog, reminders for completing modules, cognitive behavioral therapy feedback regarding goal setting, technical issues/trouble shooting, and other emails [14].

After completing the final eReCog module, program satisfaction was measured using a 4‐item online survey. Items assessed satisfaction with the program, perceived improvements in cognitive performance, likelihood of recommending the program to others, and an overall rating of the program. The latter had 10 response levels ranging from “1” (*very poor*) to “10” (*excellent*), the remainder had five response levels with verbal labels appropriate for each item [14].

### 2.7 Outcomes

Recruitment and retention were the primary feasibility outcomes. Secondary outcomes included adherence to, usability of, and intrinsic motivation to engage with eReCog, as well as compliance with assessments (neuropsychological tests and patient‐reported outcome measures [PROMs] administered at baseline and follow‐up, described below). Related objectives, data sources and measures, and a priori progression criteria are summarised in Table 1.

**TABLE 1:** Feasibility outcomes, objectives, data sources and measures, and a priori progression criteria.

Outcome	Objective	Data source or measure	*A priori* progression criteria
Recruitment	Estimate the recruitment rate	Operational data	≥ 3 participants/month during the active recruitment period
Retention	Estimate the retention rate	Operational data	≥ 80% of enrolled participants complete the trial
Adherence	Evaluate adherence to eReCog	Operational data	≥ 75% of eReCog modules completed by intervention participants after 8 weeks
Usability	Evaluate the usability of eReCog	mHealth app usability questionnaire (MAUQ)^a^ [1]	≥ 80% of intervention participants score ≥ 90 on the MAUQ [20]
Motivation	Evaluate intrinsic motivation to engage with eReCog	Intrinsic motivation Inventory (IMI)^a^ [2]	≥ 80% of intervention participants have a neutral or positive score (> 4) on all IMI scales
Compliance	Evaluate compliance with assessments^b^	Operational data	≥ 80% of enrolled participants have evaluable neuropsychological test and patient‐reported outcome data

^a^ Administered to intervention participants at follow‐up only.

^b^ Control and intervention participants.

Preliminary efficacy evidence on measures likely to be used in a definitive trial was also sought. Measures included neuropsychological tests recommended by the International Cognition and Cancer Task force (ICCTF) [3] and a suite of relevant PROMs [21, 22, 23, 24]. Specifically, neuropsychological tests (*domains of cognition* assessed by each test in parentheses) included the Hopkins Verbal Learning Test‐Revised (HVLT‐R; alternating forms 5 and 6, *learning and memory*) [25] Controlled Oral Word Association (COWA; alternating forms PRW and CFL, *verbal written fluency*) [26], Stroop Color and Word Test and Trail Making Test Part B (SCWT and TMT Part B; respectively; *executive function*) [27, 28], Trail Making Test Part A (TMT Part A; *speed of information processing*) [28], and the Wechsler Adult Intelligence Scale‐Revised Digit Span (WAIS‐R Digit Span; *attention/working memory*) [29]. PROMs included the Patient‐Reported Outcomes Measurement Information System (PROMIS) Cognitive Function‐Short Form 8a (PROMIS‐CF) [21], the European Organisation for Research and Treatment of Cancer (EORTC) Cancer‐related fatigue module (EORTC QLQ‐FA12) [22], and PROMIS Emotional Distress‐Depression‐Short Form 8b and Emotional Distress‐Anxiety‐Short Form 7a [23, 24]. More complete details of each test and measure are provided in Supporting Information S1: Table S1.

### 2.8 Statistical Methods

The target sample for this pilot trial was 38 patients. The target was largely pragmatic, based on available funds and study timeframes (i.e., a recruitment period of no more than 12 months), as well as an estimated dropout of 20% by the follow‐up assessment. Like most pilot trials, it was not powered to assess efficacy. More details on sample size determination can be found in the published protocol [15].

Analysis included all available data and was performed in R (reference index version 4.4.3). Descriptive statistics (means and standard deviations, medians and interquartile ranges, ranges, and counts and percentages) were used to summarise baseline characteristics of trial participants by study arm. Recruitment data was summarised using a rate and 95% confidence interval using the Poisson distribution. Adherence, retention, usability, intrinsic motivation, and compliance with study measures were summarised using a proportion and 95% confidence interval using the Wilson method. Data on engagement with eReCog, interactions with the facilitator, and program satisfaction were also summarised using descriptive statistics.

Neuropsychological tests and PROMs were scored using standard methods (see Supporting Information S1: Table S1). Scores from neuropsychological tests and PROMIS measures are standardised scores, or T‐scores (*M* = 50, SD = 10). Scores from the EORTC cancer‐related fatigue measures are raw scores. Descriptive statistics (means and standard deviations) were used to summarise scale scores at each assessment by study arm. Differences between the mean change scores of each study arm were estimated using analysis of covariance, adjusting for relevant baseline scores. Partial eta squared (η^2^) was calculated as a measure of effect size using the “emmeans” package and interpreted as follows: 0.01, small effect; 0.06, medium effect; and 0.14, large effect [30]. In the absence of accepted minimum important differences in the target population, the intervention was considered “promising” if differences favored the intervention arm *and* partial eta squared was 0.06 or more [31]. Analysis of covariance results were also considered but interpretation was tempered by the fact that low power adversely affects the likelihood that study findings reflect a true effect [32].

## 3 Results

### 3.1 Study Profile

**FIGURE 1:**
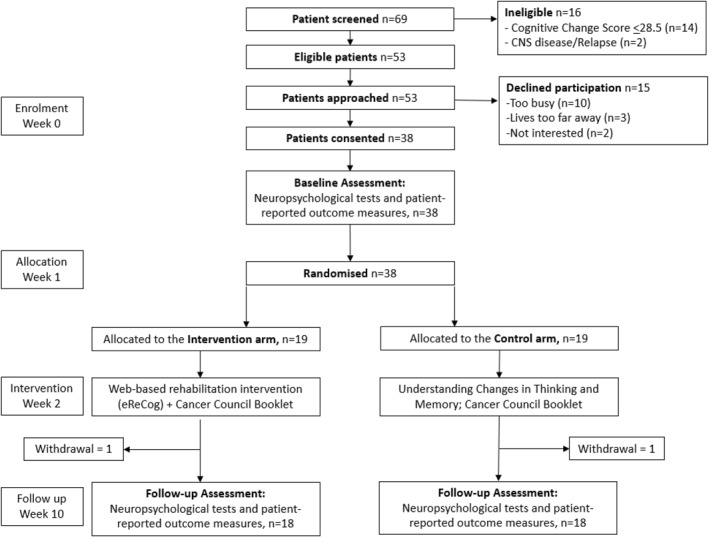
Participant flow diagram.

Screening and recruitment took place between 31 July 2023 and 27 May 2024. During this time, 69 individuals were screened for eligibility: 16 were ineligible and 53 were eligible and approached. Reasons for ineligibility are provided in Figure 1. The main reasons for ineligibility were not meeting the “cut‐off” for perceived reduction in cognitive function based on the Cognitive Change Screen [16] or had CNS disease or relapse at screening. Reasons for declining participation included living too far away, not interested and being too busy to participate. A total of 38 individuals (71%, 95% CI [59, 83]) consented to participate and 19 were randomised to each arm. Age, gender and years of education were similar between the two groups. Median days from treatment completion differed with 361 days in the control arm and 177 days in the intervention arm. Demographic and clinical characteristics of the study sample are summarised in Table 2.

**TABLE 2:** Baseline characteristics of the 38 trial participants by study arm.

Characteristic	Control arm		Intervention arm	
	(*n* = 19)		(*n* = 19)	
	*n*	%	*n*	%
Age at enrollment, in years				
Mean (SD)	53 (14)		47 (14)	
Median (IQR)	50 (42–67)		46 (30–61)	
Range	30–76		29–74	
Sex				
Male	11	58	10	53
Female	8	42	9	47
Country of birth	13	68	15	79
Australia				
Other	6	32	4	21
Language spoken at home				
English	19	100	19	100
ECOG performance status				
0	18	95	18	95
1	1	5	1	5
Marital status				
Married/De‐facto	14	74	15	79
Separated/Divorced	3	16	1	5
Single	1	5	3	16
Widowed	1	5	0	0
Years of formal education				
Mean (SD)	14 (3)		15 (2)	
Median (IQR)	14 (12–17)		16 (13–18)	
Range	9–23		11–23	
Medication for depression, anxiety, psychiatric, or neurological condition				
No	12	63	19	100
Yes	7	37	0	0
Diagnosis				
Anaplastic large cell	2	11	0	0
DLBCL	7	36	7	36
HL	8	42	10	54
Peripheral T‐cell	0	0	1	5
Transformed Follicular lymphoma	2	11	1	5
Chemotherapy regimens				
1	14	74	15	19
2	2	10	1	5
3	3	16	2	10
4	0	0	1	5
Autograft				
No	14	74	16	84
Yes	5	16	3	16
Radiation therapy				
No	14	74	12	73
Yes	5	16	7	37
Days since treatment completion				
Mean (SD)	526 (446)		252 (244)	
Median (IQR)	361 (139–947)		177 (47–378)	
Range	39–1514		28–401	

### 3.2 Feasibility Outcomes

#### 3.2.1 Primary Outcomes

As detailed above, 38 of 53 eligible individuals consented to participate in 10 months (3.8 patients/month, 95% CI [2.7, 5.2]) and 36 of 38 enrolled participants (95%, 95% CI [83, 99]) completed the trial (Figure 1). Participants who did not complete the trial were lost to follow‐up with no explanation. Recruitment and retention rates exceeded a priori progression criteria (Table 1).

#### 3.2.2 Secondary Outcomes

Results for all four secondary outcomes also exceeded a priori progression criteria (Table 1). In this case, 18 of 19 intervention participants (95%, 95% CI [87, 98]) completed ≥ 75% of eReCog modules after 8 weeks (adherence); 18 of 19 intervention participants (95%, 95% CI [75, 99]) scored at least 90 on the MAUQ (usability); 16 of 19 intervention participants (84%, 95% CI [62, 94]) had a neutral or positive score (> 4) on the IMI subscales (motivation); and 36 of 38 trial participants (95%, 95% CI [83, 99]) had evaluable neuropsychological test and PROMs data (Figure 1).

### 3.3 Engagement With eReCog and Program Satisfaction

Engagement with the program across all four eReCog modules was calculated using the engagement points listed in Table 3. On average, total participant engagement with eReCog program was 86% (*M* = 60.7 out of 76 points, SD = 6.4) and engagement with homework content was 99% (*M* = 7.9 out of 8 points, SD = 0.05). Engagement with the group discussion content was low; on average, 51% (*M* = 7.1 out of 15 points, SD = 1.2) of activities were completed. Mean completion time from initial access to Module 1 to the completion of Module 4 was 29.7 days (SD = 3.4). This is in line with the recommended completion time of 28 days [14].

**TABLE 3:** Intervention participants engagement points with “responding to cognitive concerns” by module.

	Allocated points		Actual points		
		M (SD)	% Completed	Median (IQR)	Range
Completion (days)		29.7 (3.3)		28 (28–29.5)	28–39
Module 1 (40 pages)					
Defining cognition	1	1.0 (0.0)	100	1 (1–1)	1–1
Group discussion	3	1.9 (1.4)	64	3 (0–3)	0–3
Goal setting	7	6.5 (1.4)	93	7 (7–7)	1–7
Problem‐solving	8	6.7 (2.2)	84	8 (6–8)	0–8
Relaxation training exercise	1	1.0 (0.0)	100	1 (1–1)	1–1
Total engagement	20	17.2 (3.2)	86	18 (16–19)	6–20
Module 2 (30 pages)					
Homework	3	2.9 (0.2)	98	3 (3–3)	2–3
Defining memory and impact	2	2.0 (0.0)	100	2 (2–2)	2–2
Group discussion	4	2.1 (1.9)	52	2 (0–4)	0–4
Strategies and application	11	10.8 (0.5)	98	11 (11–11)	9–11
Total engagement	20	17.8 (2.1)	89	18 (16–20)	13–20
Module 3 (18 pages)					
Homework	2	2.0 (0.0)	100	2 (2–2)	2–2
Attention exercise and feedback	4	3.9 (0.2)	98	4 (4–4)	3–4
Group discussion	5	1.9 (2.0)	38	1.5 (0–4)	0–5
Applying strategies	2	1.9 (0.2)	93	2 (2–2)	1–2
Total engagement	13	9.8 (1.9)	75	9 (8–12)	8–13
Module 4 (27 pages)					
Homework	3	3.0 (0.0)	100	3 (3–3)	3–3
Emotions and fatigue	6	6.0 (0.0)	100	6 (6–6)	6–6
Group discussion	2	1.1 (0.9)	58	2 (0–2)	0–2
Self‐care activity	6	5.6 (0.4)	94	6 (5–6)	5–6
Total engagement	17	15.8 (0.9)	93	16 (15–17)	14–17
Overall total engagement	76	60.7 (6.4)	86	63 (55–66)	45–70
Total group discussion engagement	15	7.1 (1.2)	51	8.5 (1.5–12.3)	0–14
Total homework engagement	8	7.9 (0.0)	99	8 (8–8)	7–8

The average number of emails sent between the facilitator and participants during eReCog was 17.3 (SD = 8.5) (see Supporting Information S1: Table S2). Across the different types of emails, reminders to complete activities within modules were sent most frequently (*M* = 7.0, SD = 5.3), followed by introductory emails (*M* = 3.8, SD = 0.4), and cognitive behavioral intervention feedback on goal setting (*M* = 3.5, SD = 1.3).

The overwhelming majority (95%) of intervention participants were *satisfied/strongly satisfied* with the eReCog program (see Supporting Information S1: Figure S1). Most (89%) reported an improvement in their perceived cognitive problems at the conclusion of eReCog, and 83% were likely to recommend the program to others, if they had a similar problem. Overall, 90% of intervention participants rated the program as at least *good*, approximately 41% rated it as *excellent*.

### 3.4 Neuropsychological Tests and Patient‐Reported Outcome Measures

Descriptive statistics for neuropsychological test and PROMs scale scores, along with analysis of covariance results and effect sizes are summarised in Table 4. A large effect favoring the intervention arm was observed on the SCWT Word score measuring processing speed. The difference between mean change scores was 3.7 (95% CI [0.5, 7.0]). Medium effects favoring the intervention arm were observed on the PROMIS Emotional Distress‐Anxiety‐Short Form 7a and Cognitive Function‐Short Form 8a, HVLT Total recall, SCWT Color score, and TMT A score. A medium effect favoring the usual care arm was observed on the SCWT Interference color/word score. In all instances, 95% CIs for differences in mean change scores were wide and included zero (Table 4). Trivial to small effects were observed on the remaining measures.

**TABLE 4:** Descriptive statistics for study measures at baseline and follow‐up by study arm, analysis‐of‐covariance results, and effect size estimates.^a^

Measure	Baseline		Follow‐up		*b* (95% CI)^b^	Partial η^2^ ^c^ ^,^ ^b^
	Control	Intervention	Control	Intervention		
	M (SD)	M (SD)	M (SD)	M (SD)		
Patient‐reported outcome measures						
PROMIS anxiety	53.1 (9.8)	51.3 (11.3)	52.1 (11.2)	48.2 (9.3)	−3.6 (−7.6 to 0.4)	0.09
PROMIS depression	50.6 (8.6)	46.8 (9.7)	48.6 (11.0)	46.2 (8.5)	0.2 (−3.1–3.4)	< 0.001
PROMIS cognitive function	41.4 (9.5)	41.7 (6.2)	47.2 (7.3)	50.7 (6.3)	3.2 (−0.5–6.9)	0.08
EORTC cancer‐related fatigue	39.3 (19.9)	28.8 (20.0)	28.7 (19.5)	20.8 (13.2)	−2.4 (−9.9 to 5.1)	0.01
Hopkins verbal learning test‐Revised						
Total recall	39.2 (11.2)	37.0 (10.8)	42.6 (7.8)	45.0 (9.1)	3.9 (−1.0–8.7)	0.07
Delayed recall	40.1 (12.1)	36.1 (13.5)	41.9 (11.7)	43.3 (12.1)	4.4 (−2.2–11.1)	0.05
Retention	44.1 (12.4)	41.0 (16.1)	42.5 (12.9)	43.3 (14.0)	1.8 (−6.8–10.5)	< 0.001
Recognition/discrimination	47.6 (12.2)	45.8 (11.7)	43.5 (13.5)	41.3 (16.0)	−1.9 (−11.6 to 7.9)	< 0.001
Controlled Oral word association test						
Total letter fluency	42.0 (11.8)	38.3 (10.4)	47.0 (15.5)	45.9 (13.4)	3.2 (−1.5–7.8)	0.05
Category fluency	28.8 (8.8)	28.3 (7.5)	29.1 (8.5)	28.3 (5.9)	−0.0 (−3.5 to 3.4)	< 0.001
Total written fluency	51.4 (17.9)	45.8 (16.7)	54.9 (18.7)	52.9 (17.0)	3.1 (−1.4–7.7)	0.05
Stroop Color and word test						
Word	41.2 (10.5)	39.8 (9.4)	38.9 (8.7)	42.6 (8.5)	3.7 (0.5–7.0)	0.14
Color	37.7 (9.4)	38.7 (7.3)	39.3 (9.2)	43.7 (7.6)	2.8 (−0.1–5.7)	0.10
Color/Word	47.0 (8.6)	47.4 (8.0)	48.4 (10.1)	50.1 (11.0)	0.1 (−5.0–5.2)	< 0.001
Interference color/word	50.2 (6.9)	51.1 (5.7)	54.3 (9.1)	51.3 (8.4)	−3.9 (−8.4 to 0.5)	0.08
Trail Making test						
A score	46.5 (10.7)	49.5 (11.9)	48.8 (11.0)	56.0 (10.5)	4.7 (−0.7–10.1)	0.08
B score	47.8 (10.7)	52.1 (10.0)	50.2 (10.2)	57.8 (12.3)	4.1 (−2.0–10.1)	0.05
Digit Span WAIS						
Digit span total	55.2 (10.9)	55.5 (11.0)	56.7 (9.4)	56.0 (14.0)	−1.3 (−5.6 to 3.1)	< 0.001

^a^ All PROMIS measures and neuropsychological test scores are T‐scores. The EORTC Cancer‐related fatigue scores are raw scores. For HVLT alternating forms 5 and 6 were used; for COWA alternating forms PRW and CFL were used.

^b^ Coefficient *b* provides an estimate of the difference between the mean change scores of each study arm (n.b., study arm coded as 1 for intervention and 0 for control).

^c^ Partial η^2^ is interpreted as follows: 0.01, small effect; 0.06, medium effect; and 0.14, large effect.

## 4 Discussion

Our pilot study showed promising findings of feasibility and acceptability of methods and procedures for trialling a web‐based cognitive rehabilitation program for people who have received chemotherapy for aggressive lymphoma. Our recruitment and retention rates, compliance with assessments and favorable changes on potential outcome measures suggest eReCog is acceptable to the study population.

Recruitment and retention at follow‐up assessment (primary outcomes) exceeded our expectations. Thirty‐eight people who had completed chemotherapy for aggressive lymphoma and self‐reported a reduction in cognitive performance were recruited over 10‐month, with only two withdrawing. Adherence to the eReCog intervention was very high, despite literature highlighting challenges with web‐based interventions, often demonstrating low levels of engagement and high rates of attrition [33, 34, 35]. Furthermore, attrition rates approaching 40% have been reported in previous web‐based intervention studies with cancer survivors [34], where participants stopped and/or were lost to follow‐up. This issue is one of the fundamental characteristics and methodological challenges in the evaluation of eHealth applications [35]. High adherence rates with eReCog may be due to the nature and uniqueness of the intervention. The program offered participants strategy and skill development on multiple aspects, not just cognition, but also fatigue and emotional wellbeing which are often experienced as challenges in conjunction with CRCI.

Usability of, motivation to engage and compliance with study assessments also exceeded expectations. Engagement with the intervention may be an important enabler, explaining positive outcomes of the intervention [14, 36]. Each module within eReCog contained several pages whereby participants could voluntarily enter responses to questions and activities. Across all four modules, participants' total engagement was 86%. This engagement score was similar to Mihuta et al.’s (2018) study in breast cancer survivors, where total engagement was 87% [14]. Engagement with questions pertaining to homework tasks at the beginning of modules 2, 3 and 4 was also excellent. Participants self‐reported their skills practice each week at the start of the next module with a newly learned skills rate of 99%. This rate was higher than the previous eReCog study of 87%. Implementing a method that measures the enactment of newly learned skills is critical as skills practice reinforces knowledge or behaviors related to the outcome of interest [14].However, engagement with group discussion was only 51%, again similar to the previous eReCog study (56%) [14]. Poorer engagement with group discussion activities may be due to reduced opportunities for participants enrolled at the start of the study, as they may feel more inclined to engage in group discussion if there are already responses to interact with. Finally, participants stated being satisfied or strongly satisfied with the eReCog program and nearly all rating it at least good, with nearly half rating it as excellent. Most participants perceived their cognitive problems had improved at the conclusion of eReCog and most were likely to recommend the program to others if they experienced similar problems. Similarly, other studies have shown higher adherence to, and engagement with web‐based interventions in people with cancer when they perceive ease of use, enjoyment, and usefulness of the intervention positively [37].

Several enabling factors have been associated with higher participant engagement with web‐based interventions, including a facilitator who can provide personal feedback and offer professional guidance and support [14, 38]. In our study, eReCog participants received on average 17 emails from the facilitator, including introduction emails, feedback, and professional support on module activities, reminder emails to complete necessary tasks, and follow‐up emails if tasks were incomplete. This email correspondence is similar to Mihuta et al., (2018), where an average of 21 emails were sent per participant [14]. Our high engagement scores with eReCog may be explained by the “supportive accountability” model, which suggests participants are more likely to engage with a web‐based intervention if accountable to another person [14, 33, 39]. The email exchanges between the facilitator and participants most likely contributed to high levels of engagement with eReCog, as the feedback may have contributed to participants feeling valued and their cognitive symptoms recognised. A meta‐analysis of 52 studies of 4885 healthy older adults, found web‐based interventions unsupervised or unsupported by a facilitator were less effective. The authors recommended online facilitation to enhance technology support, provide reminders and motivation cues, and to overall support adherence to the program [40]. As stated previously, the current pilot trial was not powered to assess efficacy. Potential efficacy evidence was sought but findings must be interpreted cautiously because underpowered studies have a reduced chance of detecting true effects [32, 41]. The differences in median days from treatment completion between the two groups despite being present, had little bearing on the feasibility outcomes acknowledging that these differences were purely due to chance because participants were randomised to study arms. If this had bearing on implications for potential efficacy, this aspect of the study was a secondary objective, and relevant findings are interpreted with caution [20]. That being said, findings on a number of neuropsychological tests and PROMs were considered “promising.” A large effect favoring the intervention arm was observed on a measure of processing speed (SCWT Word score). Medium effects, also favoring the intervention arm, were observed on measures of verbal learning and memory (HVLT Total recall), and processing speed (SCWT Color score and TMT A score). Finally, medium effects, also favoring the intervention arm, were observed on anxious symptomatology (PROMIS Emotional distress‐Short form‐Anxiety), and perceived cognitive functioning (PROMIS Cognitive Function‐Short Form 8a). These improvements showing promise in cognitive symptoms, cognitive performance, and anxious symptomatology warrant investigation in an appropriately powered trial.

This pilot trial has several strengths. We were able to successfully recruit and retain an understudied population with high rates of cognitive impairments.^6 11^ The sample only included people who had received chemotherapy for aggressive lymphoma and over half were male. To date, most studies that have investigated the impact of cognitive rehabilitation in oncology have only included female breast cancer survivors [12]. The use of objective and subjective cognitive measures, along with other relevant outcomes is another strength.

### 4.1 Limitations

Nevertheless, the current trial has several limitations, which may limit the generalisability of our findings beyond the study population. Participants were recruited from a single institution. They had to be proficient in English because the online version of “Responding to Cognitive Concerns” is only available in English. No measure of premorbid intelligence was administered. While not directly reflecting premorbid intelligence, the number of years of education was reported, which reflects educational attainment and is commonly reported in the literature as a proxy for IQ [20]. Finally, follow‐up was limited to approximately 8 weeks post‐randomisation, which would support the assessment of short‐term effects. As such, retention at longer‐term follow‐ups, which may be of interest to assess program effects at later points in time, is unknown.

## 5 Clinical Implications

The online version of “Responding to Cognitive Concerns” has the potential to provide better support for people affected by cancer who are self‐reporting cognitive challenges by teaching strategies to help them return more smoothly to activities of daily living, social interactions, and work, thus improving their quality of life. Adherence to, usability of, and motivation to engage with eReCog exceeded expectations suggesting that eReCog is acceptable in people with low perceived cognitive function after receiving chemotherapy for aggressive lymphoma. Persistent changes in cognitive performance are frequently reported by people affected by aggressive lymphoma, however, there is an absence of evidence‐based cognitive rehabilitation programs to support people experiencing the problem. Participants who received eReCog reported the intervention was useful, they were engaged and motivated to complete all four modules and most reported improvements in perceived cognitive abilities. While confirmation from a definitive trial is needed, this pilot found promising results for multiple domains of cognition including executive functioning, verbal learning and memory, processing speed as well as anxious symptomatology and perceived cognitive functioning. A web‐based intervention has the advantage of allowing individuals to self‐manage their care and has the potential to provide a new therapeutic option for people affected by cancer experiencing cognitive symptoms.

## 6 Conclusion

Our pilot study showed promising findings of feasibility and acceptability of methods and procedures intended for use in a definitive trial of a web‐based cognitive rehabilitation program for people who have received chemotherapy for aggressive lymphoma. Web‐based interventions have the potential to increase equity of access to cognitive rehabilitation to improve cognitive outcomes for people experiencing CRCI. Findings from this study will inform the design and conduct of an appropriately powered large‐scale trial to test the effectiveness of eReCog to improve cognitive outcomes and quality of life.

## Author Contributions

P.G. designed the study, contributed to the original concept, literature review and study design, was involved in all aspects of data collection and analysis, and the overall preparation and writing of the manuscript. K.C. was P.G.s supervisor and contributed to the original concept for this study and participated in all aspects of the design, research questions, methodology, data analysis, manuscript preparation and revision. J.G. was online facilitator for eReCog and contributed to original concept for study, methodology, data analysis, manuscript preparation and revision. KG has participated in all aspects of the design, research questions, methodology, data analysis plan, data analysis, protocol and manuscript preparation and revision. H.G., H.D., J.D., J.V., M.D., P.L. and T.D. have participated in all aspects of the design, research questions, methodology, data analysis plan, protocol and manuscript preparation and revision. H.J.G. is senior author of the eReCog intervention. All authors have been involved in drafting and critical evaluation of this manuscript. All authors have read and approved the final version.

## Funding

This study is supported by an Executive Dean Health Research Post‐Doctoral Fellowship to the first author (P.G.) provided by Deakin University.

## Ethics Statement

Ethical approval has been granted by Peter MacCallum Cancer Center and Deakin University Human Rights Ethics Committees (HREC) in Victoria Australia (HREC/97384/PMCC). This study was conducted in compliance with the principles of the Declaration of Helsinki (2013) and the principles of Good Clinical Practice and the Australian National Statement on Ethical Conduct in Human Research.

## Consent

Informed consent was obtained from all individual participants included in the study.

## Consent for Publication

Participants signed informed consent regarding publishing their data.

## Conflicts of Interest

The authors declare no conflicts of interest.

## Supporting information


Supporting Information S1

## Data Availability

The data that support the findings of this study are available on request from the corresponding author. The data are not publicly available due to privacy or ethical restrictions.
