# Supplementary material for: Web‐Based Cognitive Rehabilitation for Patients With Aggressive Lymphoma With Perceived Cognitive Impairment: A Randomised Pilot Study
Source: Psychooncology. 2026 Apr 21;35:e70469. doi: 10.1002/pon.70469 (PMC13098626; doi:10.1002/pon.70469)
Supplement: Supplementary file 1 — Supporting Information S1 [file PON-35-e70469-s001.docx]

***Supplementary Tables and Figures***

*Table S1: Details on neuropsychological assessments and PROMs*

| Name of assessment | Domain measured | What’s involved | Score interpretation |
| --- | --- | --- | --- |
| Hopkins Verbal Learning Test-Revised; alternating forms 5 and 6 | Verbal learning memory | Participants are asked to remember a list of 12 words with 3 semantic categories. Three learning trials are followed by a 24-word list in which participants are asked which words are the target words and which are distractors. | Higher scores reflect higher neuropsychological performance |
| Controlled Oral Word Association Test; alternating forms PRW and CFL | Verbal written fluency | Measures the spontaneous production of words belonging to the same category or beginning with the same designated letter. | Higher scores reflect higher neuropsychological performance |
| Stroop Colour and Word Test | Speed of information processing and executive function | Based on the observation that individuals can read words much faster than they can identify and name colours | Higher scores reflect higher neuropsychological performance |
| Trail Making Test Part A and B | Speed of information processing and executive function | Participants draw lines connecting consecutively numbered circles in part A, and then in part B connect circles with consecutively numbered alternating numbers and letters. Participants are asked to do the task as rapidly as possible. | Higher scores reflect higher neuropsychological performance |
| Digit Span Wechsler Adult Intelligence Scale | Attention and working memory | Participants remember a series and repeat them in the same order or in reverse order. | Higher scores reflect higher neuropsychological performance |
| PROMIS Cognitive-Function Short Form 8a | 8-item questionnaire assessing perceived changes in multiple domains of cognitive function including mental acuity, concentration, verbal and non-verbal memory, and verbal fluency in the past week | Respondents use a 5-item Likert scale ranging from ‘1’ (not at all) to ‘5’ (very much) to rate each item. | Higher scores reflect higher levels of perceived cognitive abilities. |
| EORTC Cancer-related fatigue module (EORTC QLQ-FA12) | 12-item questionnaire assesses physical, cognitive and emotional aspects of cancer-related fatigue in the past week. | Respondents use a 4-item Likert scale ranging from ‘1’ (not at all) to ‘4’ (very much) to rate each item. | Higher scores reflect higher levels of fatigue |
| PROMIS Emotional Distress-Depression 8b | 8-item questionnaire measures the frequency of emotions such as worthlessness, hopelessness and sadness in the last 7days | Respondents use a 5-point Likert-type scale ranging from ‘1’ (never) to ‘5’ (always) to rate each item. | Higher scores reflect higher levels of depression. |
| PROMIS Emotional Distress-Anxiety 7a | 7-item questionnaire measures the frequency of emotions such as fear, stress and anxiety in the last 7days | Respondents use a 5-point Likert-type scale ranging from ‘1’ (never) to ‘5’ (always) to rate each item. | Higher scores reflect higher levels of anxiety |

Abbreviations: PROMIS, Patient-Reported Outcomes Measurement Information System; EORTC, European Organisation for Research and Treatment of Cancer.

*Table S2: Email contact with facilitator during participation in ‘Responding to Cognitive Concerns (n = 19)*

| **Email type** | **M (SD)** | **Median (IQR)** |  | **Range** |
| --- | --- | --- | --- | --- |
| Introduction | 3.8 (0.4) | 4 (4 to 4) |  | 2–4 |
| Reminder | 7.0 (5.3) | 6 (3 to 9) |  | 0–23 |
| CBT feedback | 3.5 (1.3) | 3 (3 to 4) |  | 1–7 |
| Completion | 1.0 (0.3) | 1 (1 to 1) |  | 0–2 |
| Technical | 1.2 (3.1) | 0 (0 to 0) |  | 0–13 |
| Other | 0.6 (1.2) | 0 (0 to 2) |  | 0–4 |
| **Total** | **17.3 (8.5)** | **15 (12 to 19)** |  | **8–46** |

*Figure S1: Participant satisfaction with ‘Responding to Cognitive Concerns’ (n = 18)*
